# Olecranon Apophyseal Nonunion in Adolescent Judo Players: A Report of Two Cases

**DOI:** 10.1155/2018/8256074

**Published:** 2018-12-30

**Authors:** Kohei Yamaura, Yutaka Mifune, Atsuyuki Inui, Hanako Nishimoto, Yasuhiro Ueda, Takeshi Kataoka, Takashi Kurosawa, Shintaro Mukohara, Takeshi Kokubu, Ryosuke Kuroda

**Affiliations:** ^1^Department of Orthopaedic Surgery, Kobe University Graduate School of Medicine, Kobe, Japan; ^2^Department of Orthopaedic Surgery, Shin-Suma Hospital, Kobe, Japan

## Abstract

Olecranon apophyseal nonunion is an elbow injury from overuse that affects adolescent athletes such as baseball pitchers who participate in overhead throwing sports. However, such injury is rare in collision sports. Here, we report two patients with this condition who are Judo athletes. The purpose of this report was to describe three elbows with olecranon apophyseal nonunion in two adolescent patients participating in Judo. This is a case series; the level of evidence is 4. Two 15-year-old patients were evaluated. One suffered from unilateral and the other from bilateral chronic posterior elbow pain. They were diagnosed with olecranon apophyseal nonunion, which was treated using internal fixation and bone grafting. Radiographic evidence of the apophyseal union was observed four months postsurgery. Two elbows were treated with tension band wiring, then they underwent hardware removal six months postsurgery. Both patients returned to their previous levels of activities six months postsurgery. Internal fixation using autologous bone grafting was a useful treatment for these Judo athletes with olecranon apophyseal nonunion.

## 1. Introduction

Elbow injuries frequently occur in adolescent athletes participating in overhead throwing sports [1–3]. Stress caused by repetitive motion results in elbow injuries from overuse. Pain in an elbow injury from overuse is generally localized on the medial side. Frequently occurring injuries include medial epicondylitis, avulsion fracture of the medial epicondyle, injuries to the medial collateral ligament, and ulnar neuritis [4, 5]. On the contrary, olecranon stress fracture or apophyseal nonunion are overuse injuries that occur less frequently on the posterior side of the elbow.

Waris first reported one case of an olecranon apophyseal stress fracture nonunion in young javelin throwers in 1946 [6]. Past reports on individuals with olecranon apophyseal nonunion included baseball players [7], weight lifters [8], gymnasts [9], and divers [10]. Interestingly, olecranon apophyseal stress fracture has not yet been reported in combat sports athletes. We report two adolescent Judo athletes with chronic posterior elbow pain. Olecranon apophyseal nonunion was diagnosed and treated using open reduction and internal fixation (ORIF).

## 2. Case Presentation

### 2.1. Patients

**Figure 1:**
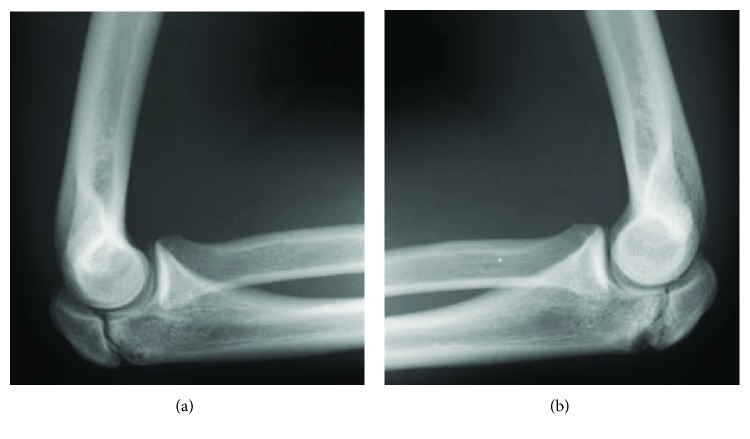
Radiographs of the elbows of a 15-year-old male patient (Patient 1). Lateral radiograph of both elbows showing delayed union of the olecranon apophysis. (a) The right elbow. (b) The left elbow.

**Figure 2:**
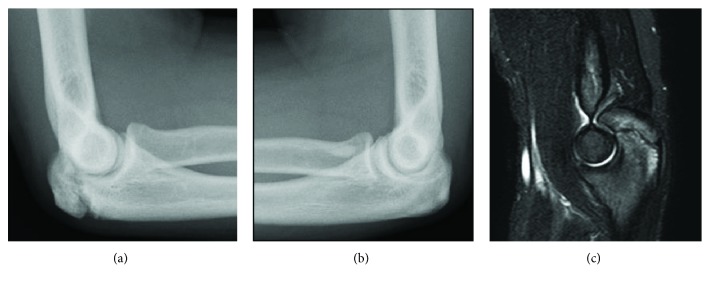
Radiographs of the elbows of a 15-year-old male patient (Patient 2). (a) Lateral radiograph of the right elbow showing delayed union of the olecranon apophysis. (b) Comparison view of the left elbow showing a completely fused apophysis. (c) Sagittal plane magnetic resonance imaging scan (short TI inversion recovery: STIR).

Two 15-year-old patients were evaluated. One suffered from bilateral (Patient 1) and the other from unilateral (Patient 2) chronic elbow pain during Judo activity as well as daily activity such as carrying a heavy bag. The symptoms lasted for 24 months in Patient 1 and 12 months in Patient 2. During the physical examination, tenderness on the posterior olecranon without limitation of the range of motion in the elbow was observed. Laxity was not identified on a valgus and varus stress test. The findings of radiographs and magnetic resonance imaging (MRI) led to the diagnosis as olecranon apophyseal nonunion (Figures 1 and 2). In Patient 1, diagnosis as apophyseal nonunion was confirmed since lateral radiographic images demonstrated the opening and sclerotic change of the olecranon apophyseal plate during its preoperative follow-up period. The MRI showed bone marrow edema around the olecranon apophyseal plate. Patient 2 was diagnosed with apophyseal nonunion since the apophyseal growth plate of the contralateral elbow was closed in the radiographic examination. Conservative treatment such as rest and avoiding Judo practice was performed for six months, which was unsuccessful. Therefore, both patients underwent surgical intervention. The preoperative Mayo Elbow Performance Scores of the three elbows of Patient 1 and Patient 2 showed 70 points.

### 2.2. Operative Technique

**Figure 3:**
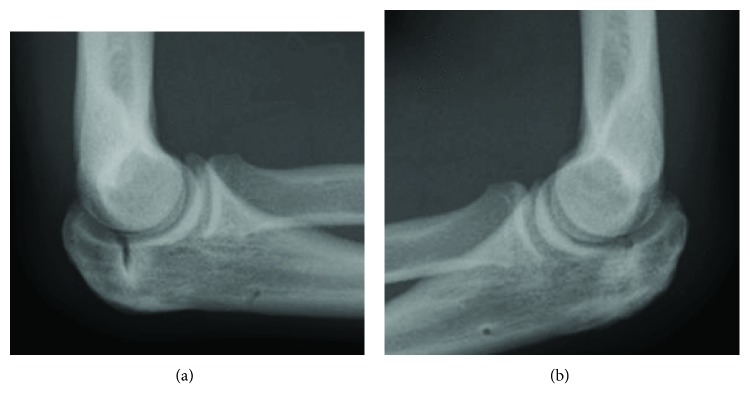
The radiographs of the elbows obtained one year after surgery. (a) Right elbow. (b) Left elbow.

**Figure 4:**
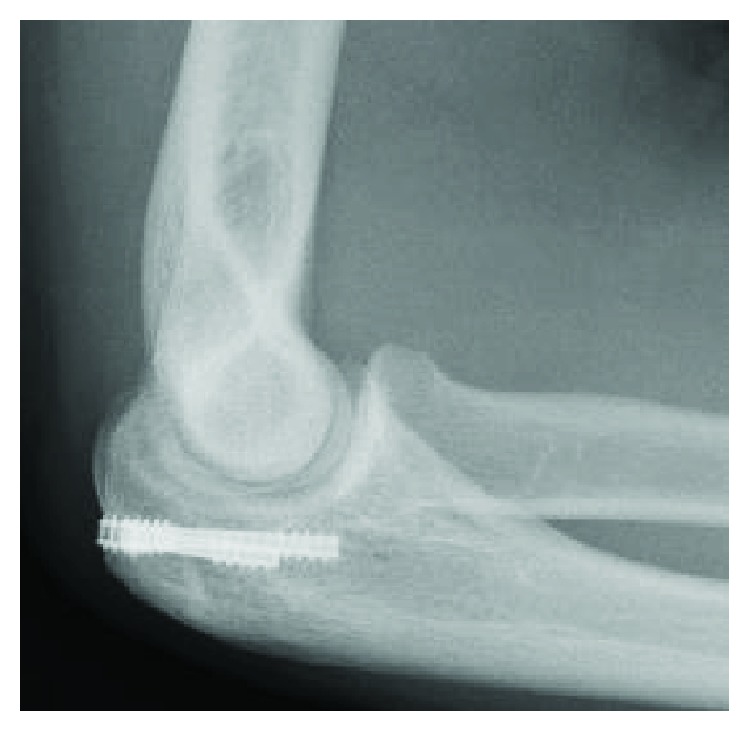
The radiographs of the right elbow one year after surgery.

A longitudinal incision over the radial aspect of the olecranon was performed 2 cm proximally and 5 cm distally from the tip of the olecranon. The nonunion site was debrided using a small sharp curette. Cancellous bone grafts harvested from their iliac crest were transplanted on the debrided site. Patient 1 underwent ORIF with K-wires and tension band wiring (Figure 3) under fluoroscopic control. Patient 2 underwent ORIF with a cannulated cancellous compression screw (DTJ standard screw; MEIRA Corp., Japan) (Figure 4). Immediately following the surgery, a 90-degree splint was adapted on the elbow for two weeks. Following splint removal, patients were allowed to perform both passive and active elbow range-of-motion exercises.

## 3. Results

Radiographic union was achieved four months postsurgery. The patient treated with tension band wiring experienced irritation from the hardware, and the hardware was removed six months postsurgery. Both patients had regained the full range of motion in their elbows and reported no pain. The Mayo Elbow Performance Scores of all the elbows one year after surgery was 100 points. The lateral radiographs of the three elbows one year after surgery demonstrated the union of the olecranon apophyseal plate. However, lateral radiographs of the right elbow of Patient 1 showed a slightly open olecranon apophysis at the final follow up (Figures 3 and 4). They returned to their previous levels of activities without any pain.

## 4. Discussion

The olecranon apophyseal nucleus appears at approximately eight years of age in females and 10 years in males, and is fused at approximately 14 years of age in females and 16 years in males [8, 11]. Olecranon apophyseal nonunion caused by overuse injury is rarer [12] compared with that in the medial side of the elbow. Torg and Moyer reported the mechanisms of this injury. They suggested that the forceful contraction of the triceps muscle causes the olecranon apophysis to separate from its apophyseal plate [2]. In addition, Rachel et al. suggested that repetitive forces can cause a stress fracture at a closed physis [13].

The majority of available literature on apophyseal fractures and the nonunion of the olecranon has discussed the possible underlying mechanisms in overhead athletes. To our knowledge, the present case series is the first report of this condition in Judo athletes. Judo is a Japanese martial art that made its official Olympic debut in 1972 for men and in 1992 for women [14]. Recently, Judo has become popular worldwide. The International Judo Federation includes over 200 affiliated countries [15]. A study evaluating French Judo athletes showed that elbow injuries account for 13.5% of all Judo injuries [16]. Specifically, for controlling the opponent, a Judo athlete has to grasp him/her by the lapel of the Judo outfit with the dominant hand (known as “tsurite”) and grasp by the hem with the nondominant hand (known as “hikite”). During both motions, an extension of the elbow is required to repeatedly and powerfully break down the opponent's posture. The mechanism underlying Judo injuries was considered to be identical to that in overhead throwing sports where a forceful and repetitive contraction of the triceps muscle was performed. The olecranon apophyseal nonunion of overhead throwing athletes is generally unilateral, and a bilateral condition is rare. Maffulli et al. reported two cases of bilateral injuries in young gymnasts [17], and Clark and McKinley reported one case of bilateral injury in a wrestler [18]. The patient with bilateral injuries is reported in this study, suggesting that the athlete repeatedly used both arms in an extended elbow position during the sports activity.

Olecranon apophyseal nonunion can be treated both surgically and nonsurgically. Schickendantz et al. reported the cases of seven professional baseball players who had a successful outcome with a nonsurgical approach based on rest and avoidance of throwing or valgus stress for at least six weeks while wearing a protective brace for four weeks. All athletes were able to return to throw at an average of eight weeks following initiation of care. Schickendantz et al. [19] and Paci et al. reported the successful outcome of the surgical treatment using tension band wiring and cannulated screw fixation [20]. In the patients presented here, nonsurgical treatment was initially performed. However, due to the lack of success of treatment for six months, surgical intervention was performed in all patients. Patient 1 was treated with tension band wiring, and the hardware had to be removed six months after surgery because the patient experienced irritation from the hardware. We treated Patient 2 with a cannulated screw, considering the avoidance of hardware removal. Both achieved successful radiographic union four months postsurgery and returned to their previous levels of activities.

In conclusion, we report the olecranon apophyseal nonunion in two Judo athletes. The athletes were treated for olecranon apophyseal nonunion with ORIF. Their clinical outcome was successful, allowing them to return to competitive Judo at their prior levels six months postsurgery.
